# Two-Flap Technique, Superior Pedicle and Central Pedicle Retro Glandular Flap: Innovative Technique to Ensure Upper Pole Fullness after Breast Reduction Surgery

**DOI:** 10.1055/s-0045-1802644

**Published:** 2025-02-10

**Authors:** Rajat Gupta, Priya Bansal, Gautam Chaudhary, Shubham Sharma

**Affiliations:** 1 Department of Plastic Surgery, Excel Hospital, C. K. Birla Hospital, Rosewalk Healthcare, New Delhi, India; 2 Department of Plastic and Reconstructive Surgery, Excel Hospital, C. K. Birla Hospital, Rosewalk Healthcare New Delhi, India; 3 Department of Plastic and Reconstructive Surgery, Consultant Private Practice, Chandigarh, India

**Keywords:** two-flap technique, breast reduction surgery, upper pole fullness, breast aesthetics

## Abstract

**Introduction:**

The two-flap technique, superior pedicle flap and central pedicle retro glandular (RG) flap, represents a novel advancement in breast reduction surgery, addressing issues such as upper pole deflation, asymmetry, and overall breast contour irregularities seen with traditional methods. This study evaluates the outcomes, efficacy, and safety of the two-flap technique, emphasizing its ability to provide enhanced aesthetic results and high patient satisfaction.

**Materials and Methods:**

A retrospective study was conducted on 165 patients who underwent the two-flap technique between February 2022 and February 2024. The technique involved preoperative skin markings for precise flap creation, using superior and central pedicles to ensure optimal vascularity and contour. Tissue reduction was performed based on individual patient needs, and post-surgical follow-ups ranged from 6 months to 1 year. Data collected included demographic details, tissue reduction volumes, postoperative complications, and patient satisfaction. Statistical analysis was conducted using SPSS software.

**Results:**

The majority of patients (66.67%) were aged 31 to 40 years, with a mean age of 34.59 years. Tissue reduction ranged from 200 to 800 g, with 48.49% of patients having 200 to 400 g removed. Complications included seroma in 7.27% and wound dehiscence in 3.64%. Patient satisfaction was high, with 89.09% reporting being “highly satisfied.” The technique demonstrated significant improvements in upper pole fullness and breast contour.

**Conclusion:**

The two-flap technique offers a safe, effective, and aesthetically pleasing solution for breast reduction surgery. Its innovative approach minimizes complications and maximizes patient satisfaction, though further studies are recommended to validate its efficacy in larger breast sizes.

## Introduction

The standards of modern plastic surgery, as articulated by Jack Penn 65 years ago, assert that enlarged breasts must be reduced to normal proportions while achieving both symmetry and aesthetic appeal. These foundational principles remain critical in contemporary practice, particularly as an increasing number of older women seek breast reduction surgery to alleviate functional discomfort and enhance aesthetic outcomes. Traditional methods, while effective in reducing breast volume, often fall short in addressing the long-term maintenance of breast shape and projection, particularly in patients of advanced age.

Historically, plastic surgeons have focused solely on nipple position during reduction mammoplasty and very little importance has been given to breast aesthetics. Photographic analysis reveals that conventional breast reduction techniques produce a linear and sometimes a concave upper pole contour. 1 The deflation of upper pole of breast following various conventional breast reduction techniques has always been a concern. Such concern was cited by Graf et al in 2000 in which she attempted to pass a dermoglandular chest wall flap under the pectoralis muscle loop in an attempt to regain upper pole fullness. Although Graff described a method involving the placement of the pedicle beneath a pectoralis muscle sling, our technique introduces unique aspects that have not been addressed in the existing literature. 2 Swanson in 2014 published a technique of breast reduction with the use of breast implants to achieve upper pole fullness. 1 But the simultaneous use of breast implants in breast reduction has been a matter of concern for various plastic surgeons all over the world.

Emerging research indicates that achieving high patient satisfaction in breast reduction surgery requires a dual focus on both volumetric reduction and aesthetic enhancement. The two-flap technique represents a significant advancement in this regard, designed to tackle common challenges associated with traditional approaches. By creating two distinct flaps from the upper and lower poles of the breast, and using the lower central pedicle as retro glandular (RG) flap, this technique provides better upper pole fullness and enhanced breast aesthetics.

Studies have highlighted the need for innovative techniques in breast reduction to improve aesthetic outcomes while reducing complications. In 2000, Graf pioneered a technique to enhance upper pole fullness by positioning a dermo glandular chest wall flap beneath a pectoralis muscle loop. 2 Similarly, in 2012, Widgerow introduced a dermal fascial suspension method, creating an “internal bra” effect to stabilize breast shape and projection. 3 These advancements illustrate the continuous evolution in breast surgery, demonstrating how innovative techniques increasingly align with both aesthetic and functional patient needs.

This article aims to present the theoretical framework, surgical methodology, and early clinical outcomes associated with the two-flap technique. Our primary objective, however, is to enhance aesthetic outcomes; by focusing on enhancing upper pole fullness and overall breast contour, this approach has the potential to improve patient satisfaction and deliver superior long-term results. Through this exploration, we seek to contribute to the ongoing advancements in breast surgery, emphasizing the significance of the two-flap technique in addressing the evolving demands of patients.

## Materials and Methods

A total of 165 patients from a retrospective cohort underwent breast reduction surgery utilizing the specified technique between February 2022 and February 2024. The study is approved by the GeneBandhu Ethics Committee (Ref- ECG030/2024); the meeting was held on October 16, 2024.

The methodology includes a comprehensive overview of the preoperative assessment and the specific steps of the surgical technique employed during breast reduction surgery.

### Marking

Skin marking is done preoperatively in the standing position. Standard breast landmarks are drawn including the sternal notch, chest midline, inframammary fold (IMF), and breast meridian. The arms may be raised to help delineate the lateral border of the breast in those with a significant excess lateral chest tissue. The Pitanguy's point, or the anterior projection of the IMF into the breast meridian, is marked by direct palpation or using a flexible ruler positioned under the breast. New nipple–areola complex (NAC) is planned over Pitanguy's point marking a circle of circumference 16 cm. 5 cm long vertical limbs are marked from the new NAC marking. The opening of this 5-cm triangle or the angle between the vertical limbs depends upon the amount of skin and breast tissue excision needed. Breast tissue is displaced superomedial to mark lateral IMF. Similarly, breast tissue is displaced superolaterally to mark medial IMF. The vertical limbs are then connected to the medial and lateral IMF marking with smooth, curvilinear lines.

**Fig. 1:**
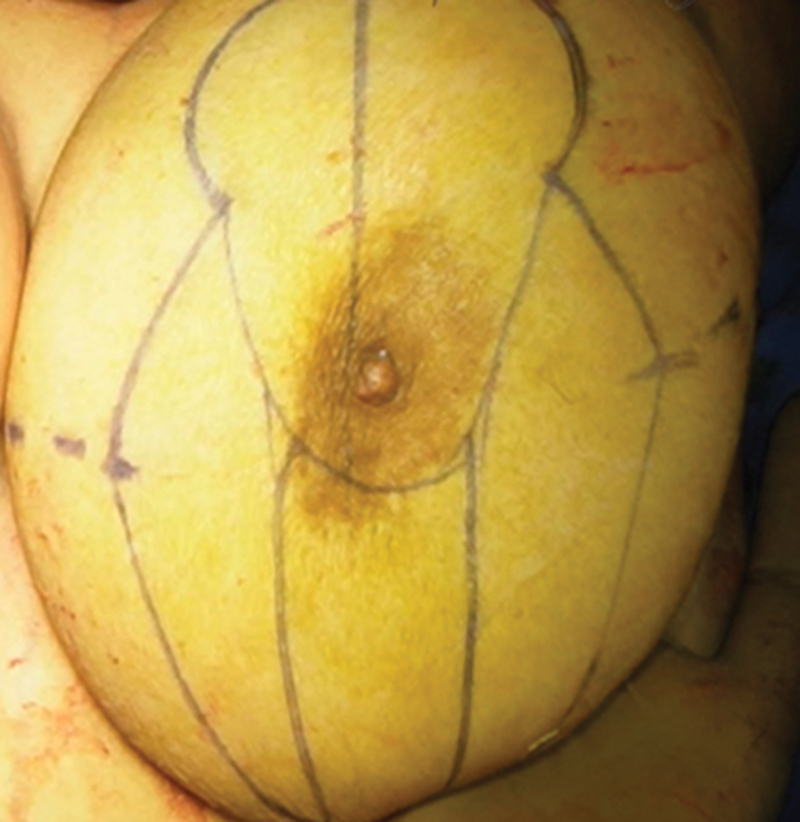
Markings for breast reduction.

The first pedicle (superior pedicle) is marked starting superiorly from the new NAC location, extending inferiorly forming a smooth U around the NAC, and terminating at the junction of junction of the new NAC with the vertical limb marking. The second pedicle (central pedicle) is marked with an approximate width of 8 cm over the breast meridian starting from the IMF and moving upwards till NAC ( Fig. 1 ).

### Surgical Technique

**Fig. 2:**
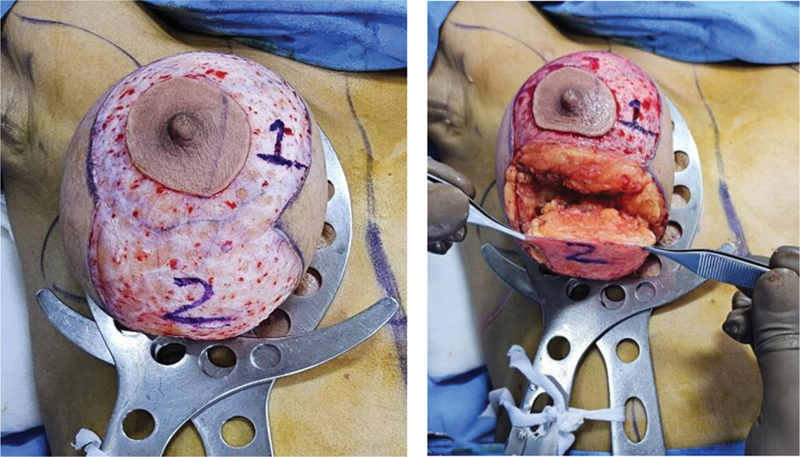
Dissection between flap 1 and flap 2.

A breast tourniquet is applied. Using an areolar marker of 44 mm, incision is marked over NAC and both pedicles are de-epithelialized ( Fig. 2 ).

**Fig. 3:**
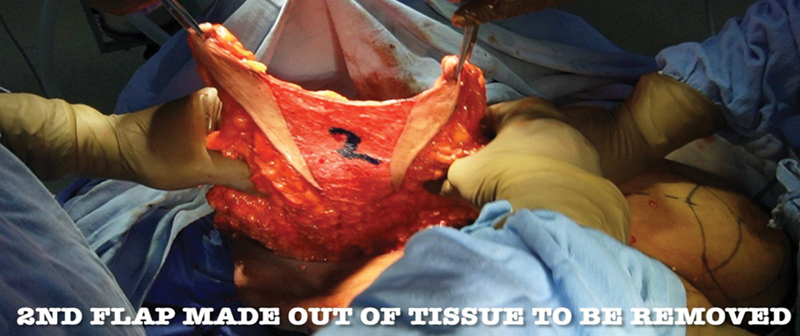
Flap 2 dissected along with lateral and medial breast tissue to be resected.

The superior pedicle (carrying the NAC) is created using the cautery to dissect straight down to the chest wall. Dissection is done with electrocautery in a perpendicular direction between the pedicles ( Fig. 3 ).

**Fig. 4:**
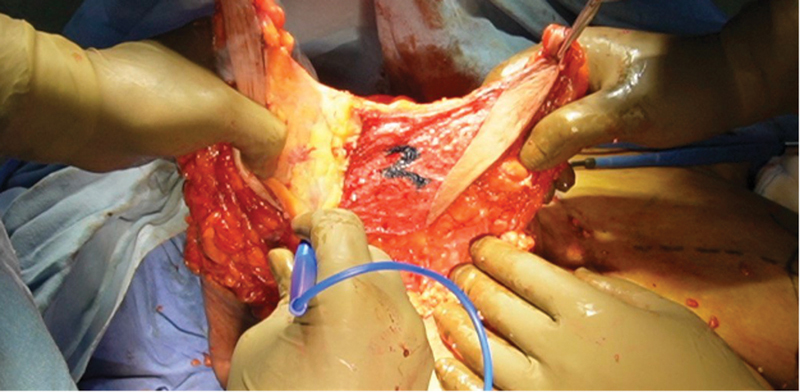
Excision of tissue lateral and medial of inferior flap 2.

The inferior breast tissue which also includes the second or central pedicle is a block of parenchyma of the lower pole of the breast. On all four sides, dissection is carried down to the pectoral fascia so that the block of tissue is totally mobile. The whole breast tissue from the lateral to inferior and then to medial extent is dissected till the pectoral fascia along with the central flap ( Fig. 4 ). Skin flap of approximate 1 cm thickness is elevated from medial and lateral aspects of the marking. Skin flap thickness at the inferior aspect is kept thinner than 1 cm, as it helps in skin re-draping at the time of skin closure.

**Fig. 5:**
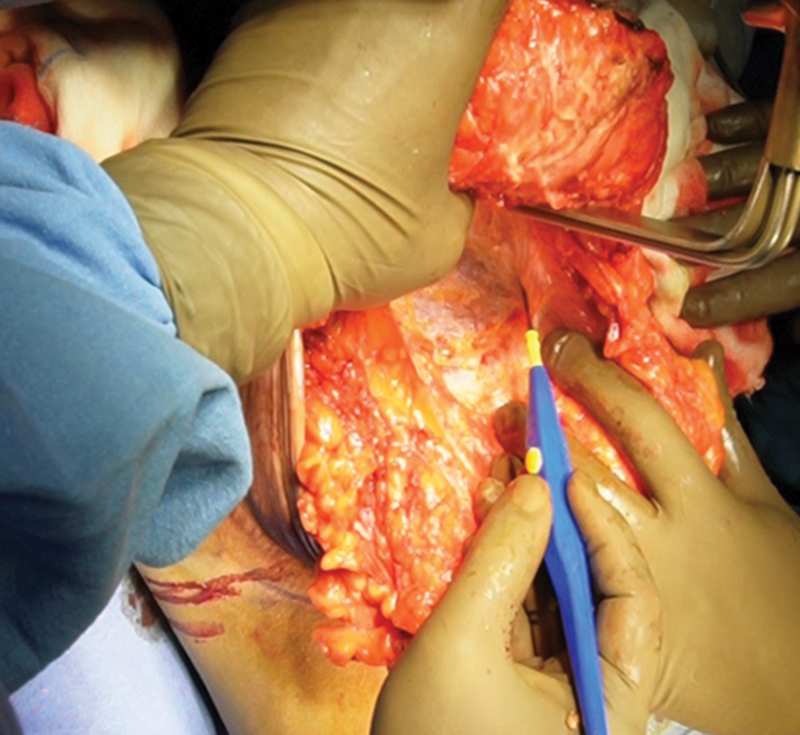
Dissecting pocket beneath flap 1/ superior flap.

Out of this whole breast tissue dissected, we remove the breast tissue medial and lateral to de-epithelized second flap, also called as central pedicle flap. This flap is based on thoracic wall perforators ( Fig. 5 ).

**Fig. 6:**
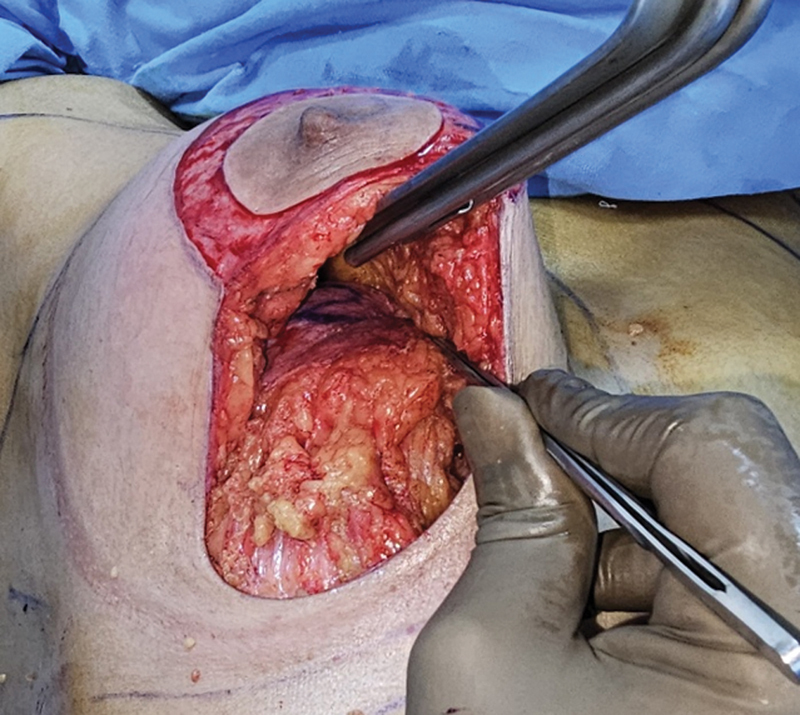
Fixation of inferior Retroglandular flap (RG) under superior flap opposite second intercostal space.

**Fig. 7:**
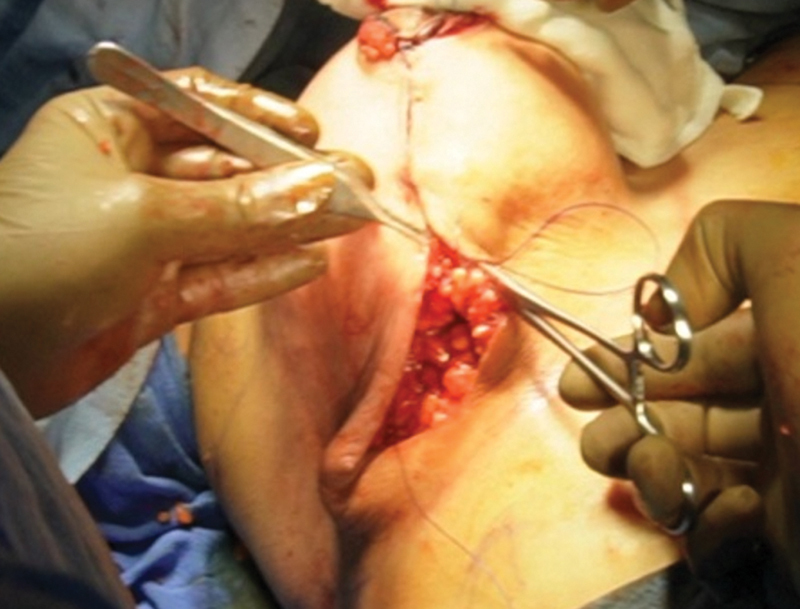
Creation of neo, IMF, Inframmamary fold.

A pocket is created under the superior pedicle (flap carrying the NAC) above the pectoral fascia up to second intercostal space ( Fig. 6 ). The second flap of dermo glandular tissue based on central pedicle is propelled under the superior breast tissue (the first flap) as RG flap and fixed to pectoralis fascia with a nonabsorbable suture (Nylon 1–0). Three to four sutures are used to fix the second flap at the upper pole opposite the second intercostal space ( Fig. 7 ).

**Fig. 8:**
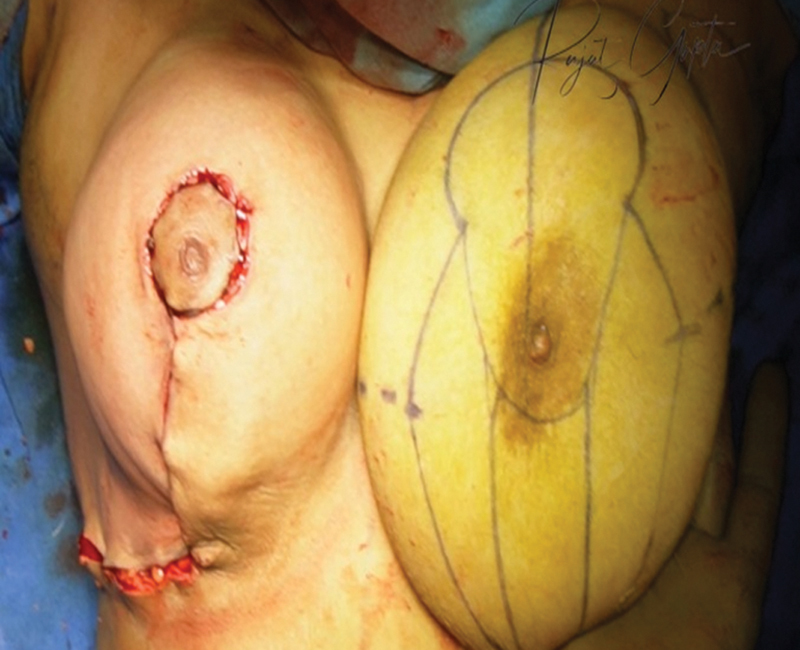
Comparison of both breasts on OT table.

The superior-most point of the vertical limbs is closed with a “2-0 PDS suture” followed by closure of vertical limbs of medial and lateral pillars. Usually, three to four sutures are used to close the pillars. New IMF is created usually at a distance of 7 to 8 cm from the NAC by suturing the lateral and medial neo-inframammary fold skin to pectoral fascia. Remaining skin incision is closed above in further two layers, Stratafix No. 1 in subcutaneous, followed by subcuticular 3–0 Monocryl ( Fig. 8 ).

Deep dermal sutures (PDS 2–0) are then used at the 12, 3, 6, and 9 o'clock positions of the NAC to set the NAC position. The remaining skin incisions around NAC is closed with 3–0 Monocryl subcuticular. No. 18 suction drains are used only in selected patients with large reductions leaving substantial dead space that required drainage. Drains were removed once the drainage was less than 30 mL/day. All patients had their drains removed within 48 hours.

**Fig. 9:**
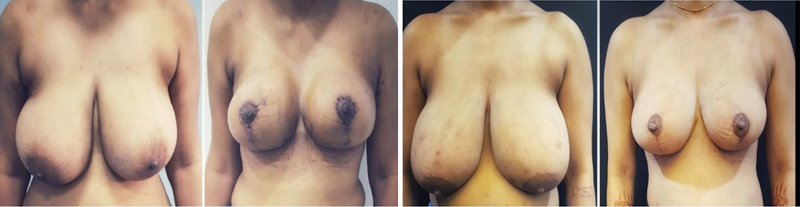
Comparison of both breasts on OT table. One can appreciate the upper pole fullness and cleavage achieved on OT table on reduced Rt breast. OT, operating table.

**Fig. 10:**
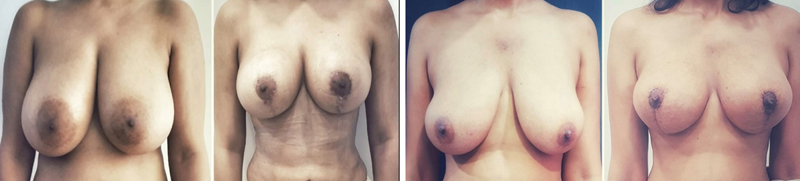
Before and after surgery of a breast reduction surgery patient using the two-flap technique. Comparison of both breasts on OT table. OT, operating table.

Data collection encompassed demographic details, final diagnoses, the volume of tissue reduced (in grams), and postoperative outcomes, including the occurrence of seromas, wound dehiscence, and patient satisfaction. Follow-up evaluations were regularly conducted up to 6 months, with additional follow-ups scheduled as necessary for any complications reported ( Figs. 9 and 10 ).

## Results

The analysis of patient data from the study provides valuable insights into demographics, surgical outcomes, and satisfaction levels. The largest age group (66.67%) was between 31 and 40 years, followed by 21.21% of patients aged 21 to 30, and 12.12% in the 41 to 50 range. The mean age was 34.59 years, with a range of 21 to 50 years ( Table 1 ).

**Table 1:** Distribution of respondents

Category	Number of patients ( *n* = 165)
**Age group (years)**	
**21–30**	35 (21.21%)
**31–40**	110 (66.67%)
**41–50**	20 (12.12%)
**Mean ± SD (range)**	34.59 ± 5.7 (21–50)
**Amount of reduction (g)**	
**200–400 g**	80 (48.49%)
**400–600 g**	55 (33.33%)
**600–800 g**	30 (18.18%)
**Presence of seroma**	
**No**	153 (92.73%)
**Yes**	12 (7.27%)
**Presence of wound dehiscence**	
**No**	159 (96.36%)
**Yes**	6 (3.64%)

Regarding marital status, 92.73% of patients were married, while 7.27% were unmarried. Tissue reduction analysis showed that nearly half of the patients had a reduction of 200 to 400 g, one-third had 400 to 600 g removed, and the remaining patients experienced a reduction of 600 to 800 grams.

Post-surgical outcomes were favorable, with 92.73% of patients not experiencing seroma, and 7.27% reporting its occurrence. Wound dehiscence occurred in 3.64% of cases.

Patient satisfaction was exceptionally high, with 89.09% rating their experience as “highly satisfied” and 9.69% as “satisfied,” indicating the procedure's success in both medical outcomes and patient experience ( Table 2 ). Satisfaction was measured using a 5-point Likert scale.

**Table 2:** Satisfaction level of the patients

Satisfaction level	Number of patients ( *n* = 165)	Percent (%)
**Highly satisfied**	147	89.09
**Satisfied**	16	9.69
**Neutral**	1	0.61
**Dissatisfied**	1	0.61
**Highly dissatisfied**	0	0

## Discussion

The present analysis provides valuable insights into patient demographics, surgical outcomes, and satisfaction levels, laying a foundation for evaluating the efficacy of the two-flap technique in breast reduction surgery. This technique has emerged as an innovative approach that offers distinct advantages over traditional methods, particularly in terms of aesthetic outcomes, minimizing complications, and enhancing patient satisfaction. By addressing key challenges such as upper pole deflation and visible scarring, the two-flap technique offers both functional and aesthetic improvements, which align with the principles of modern plastic surgery.

Maintaining upper pole fullness is a critical determinant of postoperative breast aesthetics. The two-flap technique 's innovative use of two distinct flaps of breast tissue—specifically, the superior pedicle flap for preserving the vascularity of the NAC and the central breast mound RG flap to enhance upper pole fullness—effectively addresses this challenge. The dual-flap approach contributes to a more rounded, aesthetically pleasing breast shape, a factor known to be crucial for long-term patient satisfaction. Existing literature, including studies by Gusenoff et al and Swanson, emphasizes the importance of upper pole fullness in achieving desirable breast aesthetics. 4 5 These studies reinforce the findings of this analysis, indicating that techniques that preserve upper pole contour and projection are essential for maintaining breast shape and overall satisfaction in the long term.

The low complication rates observed in this study further underscore the advantages of the two-flap technique. Wound dehiscence just accounted for only 3.64% of the total, while seroma formation was noted in 7.27% of cases. These findings are particularly notable when compared with traditional breast reduction methods, where complication rates tend to be higher. The meticulous surgical technique, careful intraoperative management, and comprehensive postoperative care protocols appear to have contributed to these favorable outcomes. The results align with previous studies such as Pusic et al and Coriddi et al, which highlight the role of advanced surgical techniques in reducing complication rates. 6 7 The remarkably low incidence of seroma and wound dehiscence in our study supports the argument that the two-flap technique represents a significant improvement over conventional approaches in terms of patient safety and recovery.

High patient satisfaction further highlights the effectiveness of this technique. In our study, 98.78% of patients expressed high satisfaction with their results. This positive feedback reflects the combined impact of improved aesthetic outcomes and functional benefits. Hidalgo noted that both functional improvements, such as relief from macromastia symptoms, and aesthetic enhancements play a significant role in patient satisfaction. 8 The two-flap technique 's ability to address both aspects accounts for the satisfaction observed in this study.

Compared with traditional methods, which often struggle to maintain upper pole fullness and tend to leave more visible scars, the two-flap technique offers clear advantages. The strategic design of this technique ensures better upper pole support using the RG flap, effectively addressing common concerns among patients seeking breast reduction surgery. Studies by Chang & Cheng and Tebbetts support the need for innovative techniques that prioritize both aesthetic and functional outcomes. 9 10 By overcoming the limitations of traditional approaches, the two-flap technique aligns with contemporary trends in plastic surgery, where achieving long-term aesthetic stability without compromising function is paramount.

The findings from this study provide strong evidence supporting the clinical efficacy of the two-flap technique in breast reduction surgery. The technique's ability to maintain upper pole fullness, minimize complications, and deliver high patient satisfaction makes it an attractive option for surgeons and patients alike.

Future research may further explore the long-term sustainability of these results and compare them across diverse patient populations to reinforce the broader applicability of the two-flap technique in clinical practice.

## Conclusion

The two-flap technique for breast reduction surgery represents a significant advancement in addressing critical challenges such as upper pole fullness and overall breast contour. The low complication rates and high patient satisfaction further validate the efficacy of this approach. However, it is important to note that this technique may be less effective for patients with larger breast sizes.

Moving forward, we believe that ongoing refinement of the two-flap technique, along with comprehensive long-term follow-up studies, will be essential in establishing it as a standard practice in breast reduction surgery. By further investigating its outcomes and patient experiences, the surgical community can ensure that this technique continues to meet the evolving needs of patients, ultimately improving their quality of life and satisfaction with surgical results.
